# Surface Anatomy of the Thorax

**Published:** 1901-07-13

**Authors:** Seymour Taylor

**Affiliations:** Physician to the Hospital


### HOSPITAL CLINICS AND MEDICAL PROGRESS.

### SURFACE ANATOMY OF THE THORAX.

Abstract of a Clinical Lecture delivered at the West London Hospital in May 1901, by SEYMOUR TAYLOR, M.D., F.R.C.P., Physician to the Hospital.

IN taking a post-graduate class over the surface anatomy and medical landmarks of the thorax, it is necessary that I should be brief, as our time is limited, and that I should mention to you certain facts which possibly may be new to you, or are at least not put forth in the usual text-books bearing on the subject; and lastly that I should not confine my observations entirely to the thorax, but that we should demonstrate certain anatomical points of interest in the neck. It will also be obvious to you that I cannot in the short hour which is allowed for our clinical lecture—for after all it is a clinical lecture—mention everything that it is necessary for you to know, but if in that time I shall have recalled to you any of the more important data which may have escaped your memory, I shall be satisfied.

*The Neck.*—In passing the finger down the middle line of the neck one is first arrested by the body of the hyoid bone. It is subcutaneous and acts as the fixed point, although far from being immobile, from which muscles radiate, which are about to move the tongue and also the apparatus concerned in deglutition, as well as that concerned in phonation; the latter of course to a slight extent only. Running down from this hyoid bone to the upper border of the thyroid cartilage, the thyro-hyoid membrane fills up the gap between these two structures. It is noteworthy inasmuch as it is traversed by a large sensory nerve (superior laryngeal) and artery going to the interior of the larynx. The thyroid cartilage, although easily felt, is only subcutaneous in the middle line. It has lateral wings, being covered by sterno-hyoid and sterno-thyroid muscles and by the lateral lobes of the thyroid body. The membrane immediately below this (the crico-thyroid) is of more importance to you surgically than the thyro-hyoid membrane, since you may be called upon to open the air-passage at this spot, and frequently this emergency comes somewhat unexpectedly, and it is well for the benefit of the patient that your fingers should have been previously exercised in discovering the whereabouts of this gap. I repeat it is easily felt on the lower border of the thyroid cartilage, having the cricoid cartilage immediately below; but the gap is a very narrow one, and will only just admit the tip of one's forefinger. We were taught in my younger days to be careful, when performing the operation of laryngo-tracheotomy, of a small artery which ran across the crico-thyroid membrane, and which might cause troublesome, or even fatal, hæmorrhage. Now I don't wish for a moment to advocate rashness and carelessness in your surgical operations, but, so far as my experience has gone, the demands which have been made by a somewhat asphyxiated patient for an immediate entry of air into his respiratory passages have been so urgent that one has not time to think of such comparatively minute risks as a hæmorrhage from the small crico-thyroid branch of artery, and one is only too glad to be able to make an incision quickly and to pass the tube into the air-pipe with success. I should, if my own life were in peril, be inclined to select some other operator than one who would be nervously dissecting down on to the membrane with a dread of this vessel before him. Below the cricoid cartilage there are about six rings of the trachea before one comes to the sternal notch. Across the second ring of the trachea the isthmus of the thyroid body lies, and you may be called upon to perform a laryngo-tracheotomy above this isthmus, about which I have spoken; or you may have to select a site below the isthmus, and in that case you will have to sever one or more of these six rings of the trachea. Here again the difficulties are not inconsiderable, and have to be remembered. Firstly, especially in children and more especially in some of the lower animals, the trachea is very mobile and may easily be pushed to one side by the pressure of the scalpel as it cuts its way through the skin. This mobility may allow, but it is difficult to think of such an accident, of the tracheotomy tube being thrust through the skin incision and not into the windpipe, although the rings may have been carefully severed, the tube itself lying to one or other side of the trachea. Another *contretemps* may occur owing to the depth of the trachea from the surface at the lower part of the neck. It is quite possible that in inexperienced hands the tracheotomy tube may be pushed into the subcutaneous cellular tissue in front of the windpipe. I need scarcely remind you that one of the first surgeons in Europe was said to have committed this error. I flatly refuse to believe for a moment that he could have done such a thing, but I have seen it done by a novice in his first attempt at the operation. Lastly, we must consider the comparatively small calibre of the windpipe in a child, the elasticity of the rings, and the possibility that a careless operator may pass the tracheotomy tube not only through the incision into the windpipe, but right through the windpipe itself into the œsophagus. I have been creditably informed that such a mistake has occurred, but in this instance the operator was unskilled and careless.

Let me now refer to the thyroid body. The connecting isthmus lies, as you know, across about the second ring of the trachea. The lateral lobes extend upwards on the side of the thyroid cartilage, very nearly to the upper borders: these lobes are not subcutaneous, but are overlapped by the sterno-thyroid, the sterno-hyoid, and to a slighter extent by the omo-hyoid muscles. Since the pathology of the disease known as Myxœdema has been made out, we are all accustomed to try to feel for the thyroid body in patients with this disorder, and we are apt to jump readily at the conclusion that because we do not feel the thyroid body therefore this organ is atrophied. I think that my sense of touch, without being unduly acute, is possibly as well developed as that of my confrères, but I have never been able to satisfy myself that I could feel and accurately map out the dimensions of a healthy thyroid body, much less those of an atrophied one. I would rather put it to you as an axiom that if you easily make out the dimensions of this gland, it is ample proof that the gland itself is hypertrophied, but, on the other hand, failure to map out its dimensions is, to my mind, no proof of the opposite condition. Its vascular supply is important. Years ago I used to tell my class that there was something in the economy of the system which was regulated by the thyroid body and which we did not understand. I used to point out that this little organ must be of the greatest importance, inasmuch as we saw the vascular system paying great attention and court to it by reason of the numerous vessels which were sent to it. The external carotid sends a branch down to it. The subclavian artery sends another tributary backwards to it; nay, even the arch of the aorta is sometimes laid under contribution, and sends a lowest branch upwards to this little and apparently insignificant structure. These three anatomical facts alone told me that the thyroid body was of great importance, although at that time I was ignorant as to what that importance might be. When you see people running about and paying attention to some personage, you naturally feel that personage is someone of importance, even although you may not know who he is or what his importance may be. The same argument holds good with the thyroid body. We see three great trunk vessels actually going out of their way, as it were, to pay tribute to the thyroid gland.

(*To be continued.*)

